# Transport Motorization and Household Food Waste in Rural China: Direct-Weighing Evidence of a Nonlinear Association

**DOI:** 10.3390/foods15152608

**Published:** 2026-07-25

**Authors:** Fangzhou Ran, Dan Zhang, Lingen Wang

**Affiliations:** 1 Institute of Geographic Sciences and Natural Resources Research, Chinese Academy of Sciences, Beijing 100101, China; ranfangzhou@126.com (F.R.); wangle@igsnrr.ac.cn (L.W.); 2 University of Chinese Academy of Sciences, Beijing 101408, China

**Keywords:** food waste, storage waste, rural households, transport motorization, food accessibility, China

## Abstract

Avoidable household food waste can arise at multiple stages of household food management, including storage, preparation, cooking, consumption, and post-cooking storage. It may also be associated with upstream food access and purchasing conditions. Using a purpose-designed 2017 survey of 204 rural households in Shandong, China, this study combines three-day household tracking with direct weighing of avoidable edible food waste. A food-purchase transport motorization index (TMI) is constructed from food-category-specific transport modes weighted by household consumption shares. Its association with observed food waste is examined using Tobit models, interval shape tests, and sensitivity analyses. TMI exhibits a nonlinear statistical association with total food waste: fitted waste increases at lower motorization levels and tends to decrease at higher levels, although formal interval tests do not confirm an inverted-U relationship for total waste. The nonlinear pattern is clearest for storage waste, whereas plate waste shows a positive linear association. TMI is significantly associated with waste occurrence, but its association with the amount wasted among households with positive waste is not statistically significant. Purchase frequency strengthens the association between TMI and waste occurrence, whereas dietary diversity shows no comparable moderating role. Associations are more pronounced for non-perishable and plant-based foods than for perishable or animal-based foods. These findings provide short-term micro-level evidence from a rural transport-transition setting.

## 1. Introduction

Household food waste is a major source of consumer-side food waste, wasting food and embedded resources and creating avoidable environmental burdens [1,2]. Recent evidence also shows that time constraints and institutional consumption settings can shape consumer-side food-waste behavior [3,4]. In China, implementation of the Anti-Food Waste Law has further increased policy and scholarly attention to waste prevention. Reducing household food waste requires attention not only to storage, meal preparation, and leftover handling after food enters the home, but also to upstream food-access conditions and purchasing organization. This upstream perspective is particularly relevant in low- and middle-income settings undergoing simultaneous consumption upgrading, market expansion, and lifestyle change [5].

Rural China provides an important transitional setting for understanding this issue. Along with rapid urbanization, rural China is experiencing profound socioeconomic transitions. On the one hand, rural households are undergoing changes in income levels, dietary structures, and consumption practices; on the other hand, improved road conditions, wider transport motorization, and stronger urban-rural market linkages are closely associated with changes in the time and space constraints of food access [6,7]. In rural areas, food markets are often distributed across villages, township markets, and county towns, so household food purchasing is constrained not only by physical distance, but also by travel time, carrying capacity, and trip scheduling. Transport motorization matters not only because it is related to higher travel speed and carrying capacity, but also because it is associated with a wider purchasing radius, more diverse food supplies, and more flexible shopping times [8], thereby corresponding to differences in households’ actual food accessibility. These differences may be related to household purchase frequency, purchase volume per trip, inventory management, and meal preparation arrangements, and may therefore be further associated with the level of household food waste.

Most research on household food waste focuses on in-home factors such as storage conditions, meal preparation, leftovers handling, and individual attitudes [9,10], while less attention has been paid to the external constraints households face before food is purchased. This gap is particularly important in rural areas, where whether households can easily reach food markets, how much food they can carry home, and whether they are able to make frequent small-batch purchases are all closely related to later inventory and consumption decisions, but these factors have not been fully incorporated into the analytical framework of household food waste. A small number of studies have examined the relationship between food access and purchasing conditions and household food consumption or waste. Summerhayes et al. (2024) find that food accessibility, retail options, and affordability are important conditions related to sustainable food consumption [11]. Using FoodAPS data, Cuffey et al. (2023) find a significant association between the retail food environment and household food waste, and this relationship is jointly related to store type, shopping frequency, and fresh-food purchases [12]. However, these studies often use the physical distance from residence to food markets or the distribution of nearby stores as proxies for access constraints, and therefore do not fully capture differences in actual accessibility across transport conditions. In fact, the same distance does not necessarily imply the same accessibility [13]. For households using different transport modes, even the same physical distance to food markets may correspond to substantial differences in actual purchasing costs, shopping frequency, and carrying capacity per trip. Measuring food-access conditions only through distance or the retail environment may therefore provide an incomplete understanding of how transport factors are associated with household food purchasing and household food waste.

More importantly, improvements in food-access conditions are not necessarily linearly associated with food waste. Belavina (2021) finds that higher grocery store density is associated with lower food waste below a certain threshold, but may be associated with higher food waste beyond that threshold [14]. Improvements in food-access conditions, including transport conditions, may be related to more frequent small-batch purchases and lower waste, but may also be related to a wider purchasing radius, larger purchase volume per trip, larger inventory size, and higher waste. Therefore, in rural areas undergoing transport and food-access transitions, the relationship between improved transport conditions and household food waste, as well as whether this relationship shows a nonlinear pattern, still needs to be examined using household-level field survey data.

Against this backdrop, this study uses household-tracking and direct-weighing data from a purpose-designed 2017 survey of 204 rural households in Shandong Province, China. We construct a food-purchase transport motorization index (TMI) and examine its association with short-term household food waste observed during the three-day survey period. A Tobit model is used as the baseline specification and is complemented by interval shape tests, two-part models, and Poisson pseudo-maximum likelihood estimation as sensitivity analyses. The analysis further distinguishes by waste stage, waste occurrence, positive waste amount, moderating roles, and food-type heterogeneity.

This study contributes to the literature in three ways. First, it shifts attention from in-home management alone to upstream food-access conditions and examines transport motorization as an underexplored correlate of rural household food waste. Second, it brings two competing channels—purchase expansion and inventory optimization—into one analytical framework to explain why transport access may be associated with nonlinear waste patterns. Third, its direct-weighing data and decomposition by waste stage, occurrence, positive amount, and food type provide short-term micro-level evidence for rural settings undergoing transport and food-access transitions.

## 2. Literature Review and Analytical Framework

### 2.1. Household Food Waste and Upstream Food-Purchase Behaviors

Household food waste is not only related to storage, meal preparation, and leftovers handling after food enters the household, but is also closely associated with planning and purchasing before food enters the household. Stefan et al. (2013) paid early attention to the role of shopping planning and daily purchasing habits in household food waste, and found significant relationships between pre-shopping planning, shopping-list use, daily purchasing routines, and self-reported household food waste [15]. Based on survey data from Danish consumers, Stancu et al. (2016) found that planning habits and shopping habits before food enters the household were both significantly related to household food waste [16]. Romani et al. (2018) systematically identified multiple factors associated with household food waste, and pointed out that pre-entry planning and purchasing behaviors are linked with post-entry storage, meal preparation, and leftovers handling, jointly shaping the level of household food waste [17].

Existing studies mainly examine pre-entry purchasing behavior from three aspects: purchasing planning, purchasing frequency, and purchasing volume. In terms of purchasing planning, the focus is mainly on whether households can arrange meals, check inventories, and make shopping lists before purchasing food. More sufficient purchasing planning is related to fewer repeated purchases and less food accumulation, and is usually associated with lower levels of household food waste [18]. Purchasing frequency is closely related to the turnover speed of household food inventories. More frequent purchasing is often associated with smaller quantities per trip and faster consumption of ingredients [19]. However, if impulse buying occurs, frequent purchasing may also increase waste. Purchasing volume determines the amount of food bought in a single trip. A larger purchase volume per trip is often associated with longer household inventory duration and a higher risk of expiration and waste.

Existing studies have discussed the relationship between pre-entry purchasing behavior and food waste in considerable detail. However, most of them mainly focus on households’ internal planning ability, shopping habits, and food management practices, while paying relatively limited attention to the constraints shaping these behaviors. Whether households can make frequent, small-batch, and demand-based food purchases is related not only to subjective planning and management ability, but also to actual food-access conditions. This is especially important in rural areas, where household purchasing frequency and purchasing volume are closely related to market distance, travel time, carrying capacity, and other factors. Therefore, based on existing research on purchasing behavior, it is necessary to further examine the relationship between food-access conditions and household food waste.

### 2.2. Transportation, Food Access, and Household Food Waste

Food-access conditions are closely related to household consumption and waste behavior. These conditions include the richness of nearby food supplies, food prices and affordability, food quality and types, store opening hours, and the convenience of reaching food markets [20].

Transport conditions are an important dimension of household food access. The convenience of reaching food markets depends not only on geographical distance but also on travel time, transport mode, carrying capacity, and shopping arrangements. Jiao and Azimian (2021) show that accessibility measures based only on spatial distance can miss important differences in actual grocery access [21]. Swayne and Lowery (2021) similarly incorporate public-transport travel time into food-access analysis and show that identical spatial distances may involve very different effective access [22]. These differences are especially relevant for rural households facing dispersed markets and limited public transport. Motorized transport may expand the feasible shopping radius, carrying capacity, and choice of purchasing time and location, whereas walking, cycling, and scheduled public transport impose different time, physical, and flexibility constraints.

Transport conditions may be related to household food waste through the organization of purchasing. Ellison et al. (2022) show that grocery-shopping convenience involves trade-offs among shopping frequency, travel costs, and waste-related behavior [19]. More flexible access may support frequent small-batch purchasing and shorter inventory duration, but greater carrying capacity may also support larger purchases and excess stocks. The relationship is therefore not necessarily one-directional and should be treated as an empirical question rather than assumed in advance.

Overall, existing studies have paid attention to the relationships between food-access conditions, transport modes, accessibility, and household food consumption and waste, providing an important basis for understanding household food waste. However, existing studies often measure food-access conditions through the spatial distance between residence and food markets, the distribution of nearby food retail outlets, and the local food supply environment, while paying insufficient attention to differences in actual accessibility associated with different transport tools. Based on this, using 3-day household tracking and direct weighing data from rural households in Shandong, China, this study constructs a purchase transport motorization index to examine the relationship between transport motorization and household food waste.

### 2.3. Analytical Framework: Purchase Expansion and Management Optimization

This study views the household as a food-purchasing and consumption unit operating under budget, time, transport, and food-access constraints. Transport motorization (M) may be associated with the organization of household food purchasing through differences in travel costs, time costs, carrying capacity, and the flexibility of shopping trips.

For analytical simplicity, household food waste (W) can be conceptually expressed as the gap between the amount of food acquired or purchased for household use (Q) and the amount effectively consumed (F). Purchase quantity is jointly associated with transport motorization and purchasing frequency (f), whereas effective consumption is mainly related to household size, dietary habits, and short-run demand fluctuation and is relatively stable over the observation period [13,23]. Accordingly, household food waste can be expressed as follows:

(1) W=Q(M,f)−F

Taking the total differential of Equation (1) with respect to transport motorization yields Equation (2). The expression motivates two competing channels through which TMI may be statistically associated with observed household food waste: purchase expansion and inventory optimization. The net relationship depends on the relative prominence of these channels. This framework guides interpretation; it does not identify causal mechanisms.

(2) $$\frac{dW}{dM}=\frac{\partial Q}{\partial M}+\frac{\partial Q}{\partial f}\times \frac{\partial f}{\partial M}$$

Transport motorization may be related to household food waste through two competing channels. First, it may be associated with a purchase expansion mechanism. As discussed in the literature on food access and purchasing behavior, lower travel costs, shorter travel time, and higher carrying capacity may be associated with households reaching more distant food markets, accessing more diverse food supplies, and bringing more food home in a single trip. This may correspond to larger purchase volume, greater food variety, and a higher possibility of unplanned or precautionary purchases. If these additional purchases exceed households’ short-run consumption needs or storage capacity, food may remain longer in household inventories and be associated with a higher risk of excess stock, spoilage, and waste [12]. Second, transport motorization may also be associated with a management optimization mechanism. With greater travel flexibility and lower costs of additional shopping trips, motorized transport may be associated with more frequent food purchases, smaller quantities per trip, and food replenishment according to actual needs. This may correspond to shorter inventory holding time, better matching between purchases and consumption, and a lower risk of storage-related food waste [18].

At lower levels of motorization, less binding food-access constraints may first be associated with a wider purchasing radius, larger purchase volume, and more food choices. In this stage, the purchase expansion channel may be more prominent and may be associated with a higher risk of excess stock, longer inventory holding time, and spoilage [24]. At higher levels of motorization, households may gradually shift from buying more to buying more on demand, so the management optimization channel may become more prominent than the purchase expansion channel [25].

This framework suggests that the relationship between transport motorization and household food waste may be nonlinear and may differ across waste stages. Storage waste is more directly linked to purchase scale, stock turnover, and inventory holding time [26], and may therefore be more sensitive to differences in food-access conditions and purchasing organization. Plate waste, by contrast, is more closely related to cooking habits, fluctuations in the number of diners, meal preferences, and perceptions of food value [27], so its relationship with transport motorization may follow a different pattern. In addition, the association between transport motorization and household food waste can be considered at two separate margins: whether a household reports any food waste and, conditional on positive waste, the amount of waste. Household purchase frequency and dietary diversity may further moderate these relationships.

## 3. Research Design

### 3.1. Data Sources and Sample Selection

This study draws on a purpose-designed survey of rural household food consumption and dietary intake conducted by the research team in Shandong Province, China, in 2017. Shandong was selected because it combines a large agricultural and rural population with substantial variation in transport infrastructure, market access, and development conditions [28]. The dataset is older than the manuscript submission year, but it has specific analytical value because it was collected for household food consumption, direct food-waste measurement, and food-acquisition behavior. The 2017 setting captures a transition stage in which rural household food access still relied mainly on offline markets and self-organized shopping trips, while transport motorization was expanding and pandemic-related disruptions had not yet occurred.

To capture heterogeneity across household endowments and location-specific conditions, the survey adopted a multi-stage stratified sampling strategy. First, considering regional economic development, urbanization level, and other relevant factors, three prefecture-level cities—Jinan, Weifang, and Dezhou—were selected from Shandong’s 17 prefecture-level divisions. Second, within each city, seven villages were selected according to their distance from the urban core, industrial structure, and development level, yielding a total of 21 villages. Third, within each village, 10 households were selected with attention to variation in income level, household structure, and other socioeconomic characteristics. The initial sample comprised 210 households.

In this study, household food consumption refers to food consumed at home, regardless of where it was purchased or prepared. The source of food acquisition did not affect its inclusion in the food-waste measurement. Food obtained through household production, gifts from others, household purchases, or other channels was classified and weighed according to the same standardized procedures if discarded during the observation period. Because the food-waste record did not retain the acquisition source of each discarded item as a separate field, food waste could not be further disaggregated by acquisition source. The analysis focuses on avoidable food waste observed during the household consumption stage, defined as edible food that could reasonably have been consumed but was discarded from household storage, immediately after breakfast, lunch, or dinner, or after being retained as leftovers but subsequently discarded without being consumed. Food discarded during preparation and cooking was not included. Inedible residues, such as vegetable peels, soybean dregs, and bones, are excluded from the definition of food waste, following Zhang et al. [29]. Household food waste is measured in two components: storage waste and plate waste. Storage waste refers to edible food discarded from household storage because it had expired or spoiled, together with meal leftovers retained after breakfast, lunch, or dinner but subsequently discarded without being consumed. Plate waste refers to edible food discarded immediately after breakfast, lunch, or dinner. Total household food waste is defined as the sum of these two components.

To ensure consistent classification across households, the research team designed a unified food-waste recording form and classification rules before the formal household survey. The recording form covered 16 major food categories, including flour and flour-based foods, rice, maize and cornmeal, beans and bean products, coarse grains, tubers, meat, eggs, milk, aquatic products, vegetables, fruits, snacks, drinks, edible oil, and condiments. Whether a discarded food item was counted as avoidable food waste was determined on site by trained enumerators according to the definition used in this study, rather than by the respondent households themselves. Inedible parts were removed before weighing. For mixed leftovers, enumerators separated edible components from inedible residues as far as possible before recording and weighing.

Food waste was measured by direct weighing. The research team provided unified recording forms, survey instructions, electronic scales, and field support, and enumerators received standardized training before entering households. Trained enumerators classified, recorded, and weighed food waste during household visits. Plate waste was recorded after breakfast, lunch, and dinner over two consecutive weekdays and one weekend day, yielding nine recorded meals per household. Storage waste, including edible food discarded from household storage and retained meal leftovers subsequently discarded without being consumed, was recorded once per day. Edible stored food was included when it was discarded during the observation window, even if it had been acquired earlier; however, the survey did not inventory all food stocks present at the beginning of the three days. The measure therefore represents short-term waste outflows observed during the survey period, not seasonal waste or a complete stockpiling cycle. Short direct-weighing windows reduce recall error and respondent burden and have been used in household food-waste research [30]. All quantitatively measured waste was converted to raw-food-equivalent weight using the survey data and the conversion methods in Li et al. [30] and Chappell [31].

In addition to the food-waste weighing records, the semi-structured questionnaire collected household characteristics, food-purchasing behavior, and food-category-specific purchasing information. For each food category, households reported purchase frequency, purchase location, distance, and the transport mode used during the previous week, with response options including walking, bicycle, e-bike, car, and public transport. These data were used to construct the TMI described in Section 3.2. The transport module covered eight purchase categories: vegetables, meat, rice, flour, corn flour, cooked wheaten foods, fruits, and drinks, and the corresponding category-specific purchasing data were used to construct the TMI. The food-waste weighing instrument served a different measurement purpose and used the 16 categories listed above. An author-prepared English translation of the questionnaire items is provided in Supplementary File S1; the 16-category waste-recording forms are provided in Supplementary File S2. Supplementary File S3 provides an author-prepared English summary of the field classification and direct-weighing guidelines applied during the 2017 fieldwork and further clarifies the relationship between the eight-category transport module and the 16-category food-waste record.

Strict quality-control procedures were implemented throughout fieldwork and data processing. Before the survey, enumerators received standardized training in food-waste classification, boundary judgment, weighing, recording, and interview procedures. During data collection, uniform electronic scales were used. Field teams consisting of trained investigators conducted household visits to complete the weighing records and questionnaire interviews. After fieldwork, the data were checked and cleaned, and the food-waste records were further reviewed by the research team. Invalid cases and observations with extensive missing information were excluded, leaving 204 valid households for analysis.

Before the formal household survey began, participating households were informed of the study objectives, the intended use of the data, and the anonymization procedures. All respondents participated on the basis of informed consent. The data were used solely for academic analysis, and all findings are reported anonymously without personally identifiable information.

### 3.2. Research Methods and Model

To examine the association between transport motorization and short-term rural household food waste, this study first constructs a household food-purchase transport motorization index from consumption shares and food-category-specific transport modes. Because observed food waste is non-negative and includes many zeros, a Tobit model is used as the baseline specification. Two-part models and PPML provide sensitivity checks. Additional analyses distinguish waste components, occurrence and positive-amount margins, moderating roles, and food-type heterogeneity. These analyses support the central question—whether TMI is statistically associated with food waste observed during the survey window—rather than establishing causal mechanisms.

#### 3.2.1. Construction of the Household Food-Purchasing Transport Motorization Index (TMI)

In rural settings, the attributes of food items are closely related to households’ purchasing strategies and transport choices. Perishable foods such as vegetables, meat, and fruit have short shelf lives and require freshness, so households are more likely to rely on walking or other light transport modes and make frequent, small-scale purchases from nearby markets. By contrast, non-perishable items such as rice, flour, and cooking oil can be stored for longer periods and are often associated with greater reliance on motorized transport with higher carrying capacity and larger but less frequent purchases [32]. As transport information for a single food item cannot fully reflect a household’s overall purchasing behavior, this study constructed the motorization index by weighting transport mode choices with each household’s food consumption structure.

Transport-mode information was collected through a semi-structured questionnaire on household food purchasing in the previous week. For each of the eight transport-questionnaire categories, households were asked to report the transport mode used to obtain that type of food, with response options including walking, bicycle, public transport, e-bike, and car.

The main transport modes used by rural households for food purchasing were classified into three levels according to carrying capacity, travel speed, and sensitivity to distance constraints, and were assigned scores of 1, 2, and 3, respectively. Food categories that were not obtained through out-of-home purchasing trips during the reference period were assigned a score of 0. The low-motorization category includes walking and bicycles, which have limited carrying capacity and rely mainly on human power. The medium-motorization category refers mainly to public transport, which provides some carrying capacity but is constrained by fixed routes and schedules, limiting flexibility. The high-motorization category includes e-bikes and cars, which are associated with greater carrying capacity and travel autonomy and are often used for larger-scale household purchasing [33,34]. In Equations (3) and (4), k indexes the eight food categories included in the transport questionnaire; accordingly, *n* = 8 in Equation (4).

The specific calculation process is as follows. For household *i*, the raw-food-equivalent weights of foods consumed within each of the eight transport-questionnaire categories across all eating occasions recorded over the three-day survey period were aggregated by food category (k) to obtain total food consumption ($Q_{ik}$). On this basis, the consumption share of each food category ($W_{ik}$) was calculated.

(3) $$W_{ik}=\frac{Q_{ik}}{\sum_{k}Q_{ik}}$$

The transport mode score ($T_{ik}$) associated with purchasing each food category was then combined with these shares to yield the household food-purchasing transport motorization index (${TMI}_{i}$).

(4) $$TMI_{i}=\sum_{k=1}^{n}W_{ik}\times T_{ik}$$

The TMI ranges from 0 to 3. A value of 0 indicates that the food consumed by the household during the survey period was not obtained through transport-assisted out-of-home food purchasing in the seven days prior to the survey, whereas higher values indicate a greater reliance on more motorized transport modes in food purchasing.

#### 3.2.2. Tobit Estimation of the Association Between Transport Motorization and Food Waste

Household food waste in the sample is strongly right-skewed and includes many zero observations. A zero means that no avoidable edible food waste was recorded for that household during the three-day window; it does not imply that the household never wastes food. The baseline specification therefore uses a Tobit model with a zero lower bound to describe the association between TMI and observed food waste. Because TMI and the waste measure differ substantially in scale, the dependent variable is defined as FW = ln(1 + total household food waste), which retains all zero observations. For interpretation, the Tobit results are reported as average marginal estimates based on the expected observed value. Because semi-continuous waste data may depart from Tobit assumptions, two-part and PPML models are additionally used to assess sensitivity to alternative distributional specifications.

The baseline Tobit specification is given by:

(5) $${FW_{i}}^{*}=\alpha_{0}+\beta_{1}TMI_{i}+\beta_{2}control_{i}+\lambda_{v}+\varepsilon_{1i}$$

(6) $$FW_{i}=\left\{\begin{array}{c} {FW_{i}}^{*},if\;{FW_{i}}^{*}>0\\ 0,if\;{FW_{i}}^{*}\leq 0 \end{array}\right.$$

where i denotes household i, and v denotes village v. ${FW_{i}}^{*}$ denotes the latent variable and $FW_{i}$ is the observed dependent variable. $control_{i}$ is a vector of household-level control variables, and $\beta_{2}$ is the corresponding coefficient vector. $\alpha_{0}$ is the constant term, $\beta_{1}$ is the coefficient of $TMI_{i}$, $\lambda_{v}$ denotes village fixed effects, and $\varepsilon_{1i}$ is the error term for household i in the baseline Tobit model.

To examine whether the relationship between transport motorization and rural household food waste is nonlinear, the quadratic term of the transport motorization index ($TMI_{i}^{2}$) is added to the baseline model, as in Equation (7).

(7) $${FW_{i}}^{*}=\alpha_{1}+\beta_{3}TMI_{i}+\beta_{4}TMI_{i}^{2}+\beta_{5}control_{i}+\lambda_{v}+\varepsilon_{2i}$$

To reduce confounding from observed household characteristics, the models include controls in three dimensions, following Everitt et al. [35] and Liu et al. [36]: household composition, economic and decision-making capacity, and external constraints and information exposure. All controls were obtained from the semi-structured questionnaire; the items used in this study are provided in Supplementary File S1. Descriptive statistics appear in Table 1. Refrigerator capacity is square-root transformed because of strong right skew and a small number of extreme values. Food-purchasing distance is constructed analogously to TMI by weighting category-specific distance bands with household food-consumption shares; zero indicates no dedicated purchase trip. Village fixed effects absorb time-invariant village-level heterogeneity. Baseline models use heteroskedasticity-robust standard errors, and Appendix D reports village-clustered and wild-cluster-bootstrap inference to address possible within-village correlation and the small number of clusters.

#### 3.2.3. Additional Analyses of Waste Components, Waste Occurrence, Waste Amount, Moderating Relationships, and Food-Type Heterogeneity

This study further examines the relationship between transport motorization and rural household food waste from three angles: waste components, waste occurrence and positive waste amount, and possible moderating relationships.

First, total household food waste is disaggregated into storage waste and plate waste. Each component is then used as the dependent variable in separate Tobit regressions to examine whether the association with transport motorization differs across waste components. As defined in Section 3.1, these two components are distinguished mainly by the process in which waste occurs. Storage waste refers to edible food discarded from household storage because it had expired or spoiled, as well as meal leftovers retained after breakfast, lunch, or dinner but subsequently discarded without being consumed. Plate waste refers to edible food discarded immediately after breakfast, lunch, or dinner.

Second, the analysis distinguishes two cross-sectional margins of observed household food waste: whether any waste is recorded during the survey period and, among households with positive recorded waste, the amount wasted. A Logit model is used for waste occurrence, and OLS is applied to the positive-waste subsample. These estimates compare households observed in different states; they do not represent within-household transitions caused by motorization. Because the positive subsample is relatively small, this analysis controls for prefecture-level city fixed effects rather than village fixed effects to avoid unstable estimation.

(8) $$\mathrm{Pr}(AnyFW_{i}^{h}=1)=F(\delta_{0}+\delta_{1}TMI_{i}+\delta_{2}TM{I_{i}}^{2}+\delta_{3}control_{i}+\lambda_{c}+\varepsilon_{3i})$$

(9) $$F{W_{i}}^{h}=\varphi_{0}+\varphi_{1}TMI_{i}+\varphi_{2}TM{I_{i}}^{2}+\varphi_{3}control_{i}+\lambda_{c}+\varepsilon_{4i},\;if\;FW_{i}>0$$

Equation (8) is the Logit model for waste occurrence, and Equation (9) is the OLS model for positive waste amount. In these equations, h denote the type of food waste, including total household food waste, plate waste, and storage waste. The dependent variable ${AnyFW}_{i}$ is a dummy indicating whether household i experiences food waste type h; it equals 1 if $F{W_{i}}^{h}>0\;$ and 0 otherwise.

Finally, this study further examines conditions under which the association between transport motorization and household food waste may differ. Two moderating variables are introduced: household food-purchasing frequency and dietary diversity. Household food-purchasing frequency (HPF) is constructed analogously to the transport motorization index, namely as a weighted composite measure based on household consumption shares across food categories. Household dietary diversity (HDDS) is measured following the FAO-recommended method, using the number of food groups consumed by the household to assign a score; higher values indicate greater dietary diversity [37].

Using household food-purchasing frequency as an example, this study first estimates an OLS model to examine the relationship between transport motorization and household food-purchasing frequency. It then adds interaction terms to a Logit model to assess whether purchasing frequency moderates the relationship between transport motorization and the likelihood of food waste. To alleviate multicollinearity, the transport motorization index and purchasing frequency are mean-centered and denoted as ${cTMI}_{i}$ and ${cHPF}_{i}$. The model specifications are given in Equations (10) and (11).

(10) $$HPF_{i}=\phi_{0}+\phi_{1}TMI_{i}+\phi_{2}TM{I_{i}}^{2}+\phi_{3}control_{i}+\lambda_{v}+\varepsilon_{5i}$$

(11) $$\begin{array}{ll} \mathrm{Pr}(AnyFW_{i}^{h}=1) & =F(\gamma_{0}+\gamma_{1}{cTMI}_{i}+\gamma_{2}{{cTMI}_{i}}^{2}+\gamma_{3}{cHPF}_{i}+\gamma_{4}{cTMI}_{i}\times {cHPF}_{i}\\ & +\gamma_{5}{{cTMI}_{i}}^{2}\times {cHPF}_{i}+\gamma_{6}control_{i}+\lambda_{v}+\varepsilon_{6i}) \end{array}$$

In addition, because foods differ substantially in shelf life, storage conditions, and consumption habits [38], the association between transport motorization and food waste may differ across food types. Accordingly, based on the baseline specification, this study further groups foods into perishable versus non-perishable foods, and animal-based versus plant-based foods, and uses subgroup regressions to explore food-type heterogeneity.

## 4. Results

### 4.1. Sample Characteristics and Key Variables

Household food waste in the sample displays a strong mass at zero, substantial cross-household heterogeneity, and a right-skewed overall distribution. Total food waste averages 178.19 g, while the median is only 7.65 g, indicating that most households waste relatively little and that the relatively high mean mainly reflects the presence of a smaller number of high-waste households. Plate waste averages 94.81 g and slightly exceeds storage waste at 83.39 g.

**Figure 1:**
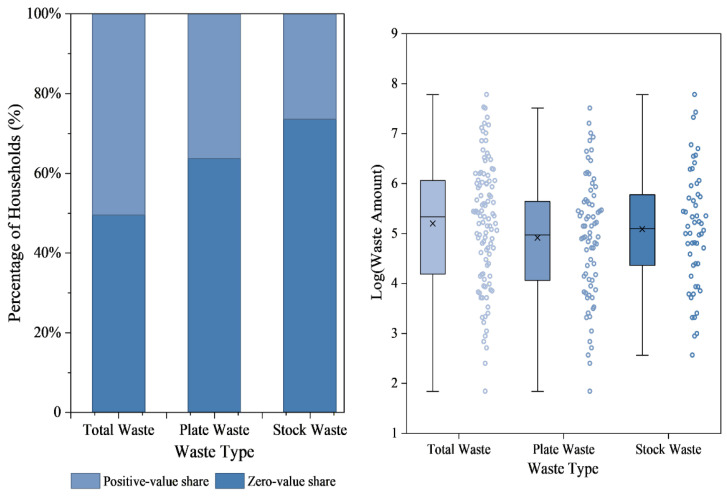
Distribution of Household Food Waste. The cross indicates the mean value.

Figure 1 shows the composition and distribution of household food waste more clearly. The zero shares for total waste, plate waste, and storage waste are 49.51%, 63.73%, and 73.53%, respectively, confirming that food waste in this study is a typical semi-continuous variable. This distribution supports the use of the Tobit model as the baseline specification and the two-part model and PPML as robustness checks.

The transport motorization index, the main explanatory variable, has a mean of 1.84, a standard deviation of 1.00, and ranges from 0 to 3, indicating substantial heterogeneity across households.

**Figure A1:**
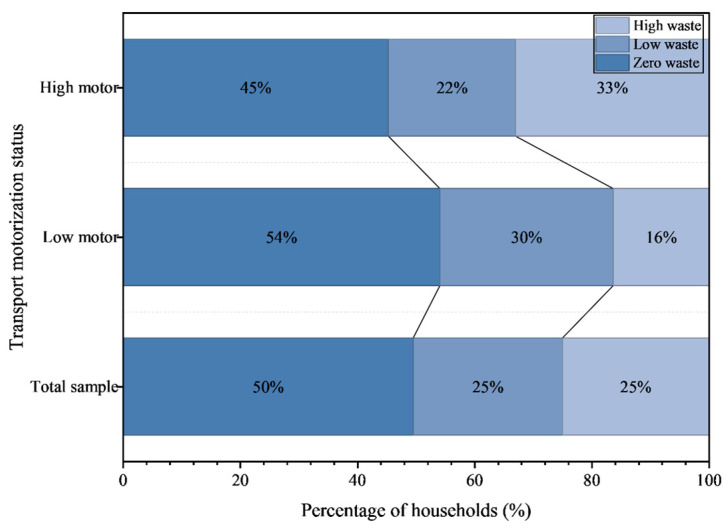
Food Waste Distribution by Low- and High-Motorization Groups.

An exploratory comparison between low- and high-motorization households, shown in Appendix A Figure A1, indicates that zero-waste households are more common in the low-motorization group, whereas high-waste households are more common in the high-motorization group. This pattern provides descriptive support for a positive association between transport motorization and household food waste.

### 4.2. The Association Between Transport Motorization and Rural Household Food Waste

Before estimating the baseline models, this study first assesses model and variable specification through correlation analysis and multicollinearity diagnostics (Appendix B Table A1). The baseline Tobit estimates are reported in Table 2. Transport motorization shows a statistically significant nonlinear relationship with rural household food waste, following a rise-then-decline pattern. The estimated turning point calculated from the coefficients is 2.53, with a standard error of 0.423 and a 95% confidence interval of [1.701, 3.360].

**Figure 2:**
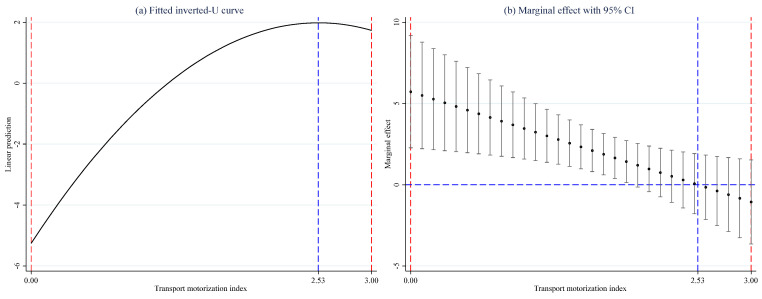
Nonlinear Association Between TMI and Household Food Waste. The red dashed vertical lines indicate the minimum and maximum observed values of the motorized transport index, the blue dashed vertical line marks the estimated turning point, and the blue dashed horizontal line in panel (**b**) indicates a marginal effect of zero.

Appendix C Table A2 reports the interval shape test. Within the observed sample range, the left-end marginal slope is significantly positive, whereas the right-end marginal slope turns negative but does not reach statistical significance. The corresponding fitted curve and marginal effect plots are shown in Figure 2. Appendix C Table A3 shows that piecewise Tobit estimates around the turning point yield the same directional pattern: the slope is positive before the cutoff and negative afterward, but the post-cutoff slope is not statistically significant at conventional levels. Taken together, these results support a rise-then-decline nonlinear pattern for total food waste, but they do not strictly confirm a formally identified inverted-U relationship.

Sensitivity analyses using alternative variable definitions, winsorization, bootstrap resampling, village-clustered and wild-cluster-bootstrap inference, and alternative estimators are summarized here and reported in detail in Appendix D.

### 4.3. Waste Stages, Waste Occurrence, Waste Amount, and Moderating Relationships

The association between transport motorization and household food waste varies across waste stages (Table 3). For storage waste, the estimates show the clearest nonlinear pattern: the linear term is positive, the quadratic term is negative, and the interval test supports a statistically significant inverted-U relationship. By contrast, plate waste is more consistent with a positive linear association.

The occurrence–positive-amount analysis reports results separately for whether any food waste is observed and for the amount wasted among households with positive observations. TMI is statistically associated with the occurrence of total food waste, plate waste, and storage waste, although the estimated patterns differ across categories. In the positive-waste subsample, the TMI coefficients are not statistically significant in the total or stage-specific OLS specifications. These cross-sectional results do not imply that the same households move from zero waste to positive waste as motorization increases.

The interaction between transport motorization and purchase frequency is positive and statistically significant in the food-waste occurrence model. By contrast, the corresponding interaction with dietary diversity is not statistically significant (Table A6).

### 4.4. Heterogeneity Analysis Results by Food Type

The association between transport motorization and rural household food waste differs across food categories. Non-perishable food waste and plant-based food waste display relatively clear nonlinear patterns, with a positive linear term and a negative quadratic term. Perishable food waste shows a weakly significant positive association, whereas the coefficients for animal-based food waste are unstable in direction and remain statistically insignificant (Table A7).

## 5. Discussion

This study finds that food waste among the sample households is characterized by a strong mass at zero, a right-skewed distribution, and substantial cross-household heterogeneity. This pattern is not unique to the present sample. Qi et al. (2020), using long-term household food-weighing data from the China Health and Nutrition Survey, found that about 40% of households reported no food waste over a three-day period and that waste amounts varied markedly across households [39]. Herzberg et al. (2020), using food-waste diary data, found that household food waste in Germany was clearly right-skewed, with many households reporting low levels of waste and some households reporting no food waste during the survey period [40]. This feature is related to the measurement approach, the definition of food waste, and the rural household context in this study. Different food-waste measurement methods may yield different results. Direct weighing generally provides relatively accurate measurements, while whether inedible parts are included is also important for the comparability of estimated waste levels [41]. This study uses direct weighing and focuses only on avoidable food waste that was originally edible but discarded at the household consumption stage. Therefore, the food-waste level reported in this study mainly reflects the actual disposal of edible and avoidable waste during short-term household tracking, and should not be directly compared with total estimates based on self-reported data, diary methods, or measures that also include inedible parts. In addition, rural households often have relatively strong frugality habits. Leftover food may be eaten later or reused through feeding livestock and poultry, composting, or other practices [42], and therefore is not counted as avoidable food waste as defined in this study.

The estimates indicate a nonlinear statistical relationship between TMI and observed rural household food waste rather than a simple linear association. For total waste, fitted values rise at lower TMI levels and tend to decline at higher levels, although the interval test does not strictly confirm an inverted-U shape. This pattern is consistent with two competing purchasing channels. At lower motorization levels, reduced travel constraints and greater carrying capacity may be associated with compensatory purchasing, precautionary stockpiling, and larger purchases; if stock monitoring and meal planning do not improve simultaneously, additional purchases may be related to inventory accumulation and avoidable waste [12,14,43]. At higher levels, greater mobility may support more flexible replenishment and shorter inventory holding times [19]. Because the data are cross-sectional and the observation period is short, these channels are plausible interpretations supported by prior literature, not directly identified causal mechanisms or evidence of within-household change.

The stage-specific results further show that the rise-then-decline pattern is mainly reflected in storage waste, whereas plate waste is more consistent with a positive linear association. Existing studies suggest that transport or shopping accessibility is closely related to the organization of household food purchasing, including shopping frequency and purchase quantity per trip [14], but it is not necessarily directly related to households’ immediate consumption capacity during meals. Compared with purchasing behavior, dietary intake appears to have a weaker relationship with distance and travel time [44]. Plate waste is also more closely associated with meal preparation habits, food preferences, and household eating patterns [45]. Therefore, the stronger statistical pattern observed for storage waste should be understood as evidence that the TMI–food waste relationship is more clearly reflected in purchase and storage-related waste than in meal-stage leftovers. This finding suggests that inventory management may be a relevant focus for household food-waste prevention in regions undergoing transitions in transport and food access.

The occurrence and positive-amount results further show that TMI is more closely associated with whether any waste is observed than with the amount wasted among households with positive observations. The statistical pattern therefore lies mainly at the boundary between zero and positive observed waste. This is consistent with evidence that food accessibility may be related to food waste through purchasing behavior [46,47], but it should not be interpreted as a motorization-induced transition within the same household.

In the food-waste occurrence model, purchase frequency shows a positive moderating relationship with transport motorization. When convenient transport and frequent shopping coexist, unplanned purchases and impulse buying may become more likely. Although some studies suggest that small-batch and frequent shopping is associated with lower food waste, this pattern is not automatic. Without shopping plans and inventory management, this strategy may also be linked to higher waste levels [48]. By contrast, the interaction with dietary diversity is not statistically supported, suggesting that eating a more varied diet does not automatically correspond to a higher transport-related food-waste risk. This pattern is also consistent with Zhang et al. (2024) [49].

The heterogeneity results suggest that transport motorization is more strongly associated with non-perishable and plant-based food waste, whereas its relationship with animal-based food waste is not statistically significant. Different food categories may be linked to different household waste-related behaviors. Shopping habits, storage practices, date-label understanding, and inventory management may be related to different types of food waste in different ways [38]. Non-perishable foods are less likely to spoil in the short term, but in contexts with improved transport conditions, they may be purchased in larger quantities and stored for longer periods. They may also be more closely associated with disposal related to forgotten stock, repeated purchasing, quality decline, or passing best-before dates [50,51]. Therefore, the relationship between broader purchase access, larger purchase quantities, and food waste may be more visible in stock accumulation and later disposal of non-perishable foods than in additional waste of perishable foods. By contrast, perishable foods have short shelf lives and require stricter storage conditions. Households may therefore tend to consume them sooner or purchase them in smaller quantities according to need. As a result, easier food access does not necessarily correspond to a statistically significant short-term increase in perishable food waste. Plant-based foods include grains, beans, tubers, and most non-perishable food items. These foods are often consumed frequently, purchased in larger quantities, and have relatively low unit values for some categories. This is consistent with the stronger association observed between transport motorization, purchase expansion, inventory management, and plant-based food waste []. Animal-based foods usually have higher prices, and their purchase frequency and storage practices are more constrained by food-safety and quality concerns [52]. Accordingly, improved transport conditions may not show an immediate or stable statistical relationship with additional animal-based food waste.

It should be noted that this study uses survey data from rural households in Shandong Province, China, in 2017, a period when rural transport and food access were still undergoing transition. Around 2017, rural transport motorization in Shandong Province was still expanding, and household food access still relied mainly on offline physical markets. This context is therefore suitable for observing the relationships among transport motorization, purchasing organization, and household food waste. Since then, the food-access environment in rural China has changed markedly. Rural roads and other infrastructure have continued to improve, rural e-commerce and logistics systems have developed rapidly, and rural household income has continued to rise [53,54]. These changes may alter the constraints and organization of household food purchasing. On the one hand, better transport and logistics conditions may be associated with lower food-access costs and more flexible purchasing arrangements [55]. On the other hand, the development of e-commerce, delivery services, and online purchasing channels may weaken the direct link between offline travel and food access, and may also change shopping frequency, purchase radius, and household inventory pressure [56]. Therefore, in regions with high levels of digitalization and well-developed delivery systems, the relationship between transport motorization and food waste may also depend on online purchasing, instant delivery, and income growth. For regions that still rely mainly on offline markets and household travel for food access, and that are undergoing transitions in transport and food access, the findings of this study on purchasing organization, inventory management, and food waste may still provide reference.

## 6. Conclusions and Recommendations

### 6.1. Main Conclusions

Using three-day household-tracking and direct-weighing data collected in 2017 from 204 rural households in Shandong Province, China, this study examined the association between food-purchase transport motorization and short-term observed household food waste. For total waste, the coefficient pattern rises and then declines, but the formal interval test does not confirm an inverted-U relationship. The clearest nonlinear evidence appears for storage waste, whereas plate waste shows a positive linear association. TMI is significantly associated with waste occurrence, but its association with the amount wasted among households with positive waste is not statistically significant. Purchase frequency strengthens the TMI–waste occurrence association, whereas dietary diversity does not show a comparable moderating role. Associations are more pronounced for non-perishable and plant-based food waste.

### 6.2. Policy Recommendations

(1) Rural transport improvement and household food-waste prevention should be considered together in settings where households are moving from constrained access to more flexible motorized shopping. Decision makers should not assume that improved access is automatically waste-reducing. Transport and market-access programs can be paired with adaptable guidance on demand-based purchasing, shopping lists, household stock checks, and first-in–first-out use so that greater accessibility does not simply translate into larger purchases and longer storage.
(2) Because the strongest statistical pattern concerns storage waste and waste occurrence, prevention should prioritize inventory-oriented measures that can be adapted to local conditions. These include stock rotation, reminders against over-purchasing, package-size options suited to household demand, and simple tools that help residents match shopping decisions with expected consumption. Meal planning, moderate preparation, and safe leftover reuse remain complementary measures for plate waste.
(3) Food-type-specific measures can complement these general principles. For non-perishable and plant-based foods, prevention should focus on excessive stock build-up, forgotten inventories, and prolonged storage. For perishable foods, demand-based purchasing and appropriate preservation remain important. Because the animal-based estimates are statistically insignificant and unstable, policy design should avoid assuming a uniform TMI–waste relationship across all food categories.

These recommendations should be treated as adaptable prevention categories for rural transition settings, not as direct prescriptions for all current rural areas. Their transferability depends on local retail access, digital purchasing, delivery services, household income, food culture, and infrastructure.

### 6.3. Research Limitations and Future Directions

This study has several limitations. First, the sample comprises 204 rural households in 21 villages in Shandong Province in 2017. The findings are most relevant to rural transport-transition settings and should not be generalized directly to other provinces, countries, or present-day rural China without considering post-2017 changes in e-commerce, delivery services, incomes, retail systems, and transport infrastructure [53,54,55,56]. Second, food waste was tracked and directly weighed for three consecutive days. This design provides relatively accurate short-term observations but cannot capture seasonal variation, holidays, market cycles, long storage periods, or persistent behavior. Initial household food stocks were not inventoried, although edible stored food discarded during the observation window was recorded; storage waste may therefore be underestimated relative to a complete inventory cycle. In addition, although own-produced food was included and the same inclusion, classification, and weighing rules were applied regardless of acquisition source, the acquisition source of each discarded item was not recorded as a separate field. The data therefore do not permit food waste to be disaggregated among household own production, purchases, gifts, and other acquisition channels, or allow source-specific waste patterns to be examined. Third, the design is observational and cross-sectional, so the results describe statistical associations rather than causal effects or within-household transitions. Future research should use longer repeated direct-weighing periods, conduct an initial household food-stock inventory, record the acquisition source of discarded food, employ panel designs, natural experiments, or policy shocks, and explicitly measure online purchasing and delivery channels.

## Figures and Tables

**Table 1:** Summary Statistics of Variables.

Variable	Mean	SD	Min	Max
**Dependent variable**				
Total household food waste	178.19	357.10	0	2398
**Explanatory variable**				
Transport motorization index (TMI)	1.84	1.00	0	3
**Control variables**				
Household size	3.90	1.78	1	10
Number of elderly members	0.91	0.93	0	3
Number of children	0.74	0.86	0	3
Health status	0.88	0.33	0	1
Household income	2.61	1.05	1	5
Educational attainment of household head	2.74	0.84	1	5
Refrigerator capacity	13.68	4.19	0	35.78
Rural type	2.14	0.83	1	3
Food-purchasing distance	1.42	0.68	0	3.31
Food-waste information exposure	2.50	1.26	1	5

**Table 2:** Baseline Tobit Estimates of TMI and Total Household Food Waste.

	(1)	(2)
Variable	FW	FW
TMI	0.727 ** (0.298)	2.557 *** (0.781)
TMI^2^		−0.505 ** (0.205)
Control variables	Yes	Yes
Village FE	Yes	Yes
Log likelihood	−339.45	−336.31
F-test	16.30 ***	17.64 ***
Pseudo R^2^	0.123	0.131
Left-censored observations	101	101
N	204	204

Note: *** *p* < 0.01, ** *p* < 0.05, * *p* < 0.1. The Tobit estimates report average marginal estimates based on the expected observed values; heteroskedasticity-robust standard errors are reported in parentheses.

**Table 3:** Stage and Occurrence–Positive-Amount Estimates of TMI and Household Food Waste.

	(1)	(2)	(3)	(4)	(5)	(6)	(7)	(8)
Variable	PW	SW	AnyFW	FW	AnyPW	PW	AnySW	SW
TMI	1.686 ** (0.762)	1.973 *** (0.690)	0.575 *** (0.166)	−0.451 (0.729)	0.421 ** (0.190)	−0.315 (0.867)	0.462 *** (0.169)	−1.178 (1.148)
TMI^2^	−0.202 (0.204)	−0.470 *** (0.167)	−0.118 ** (0.046)	0.088 (0.187)	−0.056 (0.052)	0.131 (0.217)	−0.112 *** (0.042)	0.236 (0.284)
Control variables	Yes	Yes	Yes	Yes	Yes	Yes	Yes	Yes
City FE	No	No	No	Yes	No	Yes	No	Yes
Village FE	Yes	Yes	Yes	No	Yes	No	Yes	No
Pseudo R^2^/R^2^	0.109	0.151	0.262	0.121	0.169	0.174	0.207	0.275
Overall significance statistic	Wald x^2^ = 16.84 ***	Wald x^2^ = 16.51 ***	Wald x^2^ = 51.39 ***	F = 1.42	Wald x^2^ = 34.15	F = 1.18	Wald x^2^ = 33.11	F = 1.97 **
Log likelihood	−277.10	−213.04	−95.17	-	−103.45	-	−83.63	-
N	204	204	186	103	185	74	168	54

Note: *** *p* < 0.01, ** *p* < 0.05, * *p* < 0.1. The Tobit model reports average marginal estimates based on the expected observed values, and the OLS model reports linear regression coefficients. Heteroskedasticity-robust standard errors are reported in parentheses. The pseudo R^2^ reported for the Tobit and Logit models is McFadden’s pseudo R^2^, while the OLS model reports the conventional R^2^, which represents the goodness of fit for ordinary least squares regression. The Logit model excludes some observations due to perfect prediction, and the OLS model includes only the subsample with positive waste amounts.

## Data Availability

The original contributions presented in the study are included in the article/Supplementary Materials, further inquiries can be directed to the corresponding author.

## Supplementary Materials

The following supporting information can be downloaded at: https://www.mdpi.com/article/10.3390/foods15152608/s1, File S1. Household food consumption and nutrient intake questionnaire (excerpt); File S2. Dietary consumption record form; File S3. Field classification and direct-weighing guidelines.

## Appendix B

**Table A1:** Correlation Matrix of Main Variables.

	Transport Motorization Index	Household Size	Number of Elderly Members	Number of Children	Health Status	Household Income	Educational Attainment of Household Head	Refrigerator Capacity	Rural Type	Food-Purchasing Distance	Food-Waste Information Exposure
transport motorization index	1										
Household size	0.080	1									
Number of elderly members	−0.041	0.114	1								
Number of children	0.087	0.788 ***	−0.005	1							
Health status	0.036	−0.131 *	−0.165 **	−0.028	1						
Household income	0.054	0.510 ***	−0.182 ***	0.371 ***	−0.067	1					
Educational attainment of household head	0.064	−0.002	−0.238 ***	0.041	0.009	0.125 *	1				
Refrigerator capacity	0.010	0.134 *	−0.043	0.030	0.015	0.134 *	0.067	1			
Rural type	0.265 ***	0.037	−0.067	0.005	0.100	0.007	0.004	−0.053	1		
Food-purchasing distance	0.805 ***	0.050	0.061	0.096	0.018	−0.018	−0.008	0.021	0.181 ***	1	
Food-waste information exposure	−0.010	0.058	−0.089	0.040	0.040	0.045	−0.050	0.118 *	−0.068	0.048	1

Note: *** *p* < 0.01, ** *p* < 0.05, * *p* < 0.1.

## Appendix C

**Table A2:** Turning-Point Estimates and Interval Shape Tests for Total Food Waste.

Indicator	Estimate	Statistic	*p*-Value	95% Confidence Interval
Theoretical turning point	2.531	5.98	0.000	[1.701, 3.360]
Left-end slope	5.722	3.27	0.001	-
Right-end slope	−1.062	−0.81	0.210	-
Utest	-	0.81	0.21	-
Fieller interval	-	-	-	[1.950, 5.949]

**Table A3:** Piecewise Tobit Estimates around Alternative Candidate Cutoffs.

Candidate Cutoff	Slope Before Cutoff	Standard Error	*p*-Value	Overall Slope After Cutoff	Standard Error	*p*-Value	Test of Slope Change Term (*p*-Value)
2.30	2.739	0.885	0.002	−1.047	1.579	0.508	0.066
2.40	2.596	0.866	0.003	−1.280	1.829	0.485	0.092
2.50	2.541	0.847	0.003	−1.894	2.196	0.389	0.095
2.53	2.545	0.840	0.003	−2.230	2.347	0.343	0.089
2.60	2.541	0.818	0.002	−3.183	2.758	0.250	0.074
2.70	2.500	0.773	0.001	−5.234	3.595	0.147	0.053

## Appendix D

Columns (1)–(5) of Table A4 report the results from five robustness checks: replacing the dependent variable, replacing the key variable of interest, winsorizing the dependent variable at the 1% level in both tails, conducting 500 bootstrap replications, and using village-clustered standard errors. The alternative dependent variable is per capita food waste per meal (PCFW), calculated as total household food waste during the survey period divided by the product of the average number of diners per meal and the number of meals consumed at home. The alternative measure of transport motorization is a re-estimated transport motorization index (TMI2). In this index, walking and bicycles are classified as low-motorization transport modes and assigned a value of 1, whereas electric bicycles, cars, and public transport are classified as high-motorization transport modes and assigned a value of 2. The index is then constructed using food-consumption weights across categories. Across these robustness checks, the signs and significance levels of the key coefficients remain largely unchanged, providing support for the robustness of the baseline results.

Because the sample households were drawn from only 21 villages, we further employed the wild cluster bootstrap method as a supplementary inference check. The *p*-values for the linear term of the core explanatory variable, its quadratic term, and their joint significance test were 0.008, 0.027, and 0.014, respectively. These results continue to support the robustness of the baseline pattern after inference is adjusted for the small number of clusters.

**Table A4:** Additional Robustness Checks Using Alternative Measures, Winsorization, Bootstrap, and Village-Clustered Inference.

	(1)	(2)	(3)	(4)	(5)
Variable	PCFW	FW	FW	FW	FW
TMI/TMI^2^	1.126 *** (0.376)	4.729 *** (1.623)	2.547 *** (0.780)	2.557 *** (0.968)	2.557 *** (0.835)
TMI2/TMI2^2^	−0.223 ** (0.099)	−3.359 ** (1.496)	−0.502 ** (0.205)	−0.505 ** (0.246)	−0.505 ** (0.205)
Control variables	Yes	Yes	Yes	Yes	Yes
Village FE	Yes	Yes	Yes	Yes	Yes
Log likelihood	−260.70	−337.65	−336.29	−336.31	−336.31
F-test	17.12 ***	19.46 ***	17.60 ***	-	-
Pseudo R^2^	0.165	0.128	0.131	0.131	0.131
Left-censored observations	101	101	101	101	101
N	204	204	204	204	204

Note: *** *p* < 0.01, ** *p* < 0.05, * *p* < 0.1. The Tobit estimates report average marginal estimates based on the expected observed values. Heteroskedasticity-robust standard errors are reported in parentheses for columns (1)–(4), and village-clustered standard errors for column (5).

When alternative estimation methods are considered, household food waste is treated as a two-stage process: whether waste occurs, and, conditional on occurrence, how much is wasted. We therefore first estimate a Probit model to examine the association between transport motorization and the probability of food-waste occurrence. We then estimate a generalized linear model (GLM) with a Gamma distribution and log link for the subsample of households with positive food waste, in order to examine the relationship between transport motorization and waste intensity. This specification is suitable for strictly positive and right-skewed continuous data and helps accommodate potential heteroskedasticity in positive samples to a certain extent. In the Probit model, the linear term of the transport motorization index is positive and significant at the 1% level, while the quadratic term is significantly negative. In the GLM, the signs of the key coefficients are consistent with those in the baseline model, although their statistical significance is weaker. Furthermore, considering potential heteroskedasticity in the data, we re-estimate the model using PPML. The results again show a significantly positive linear term and a significantly negative quadratic term for the transport motorization index, consistent with the baseline estimates.

**Table A5:** Alternative Estimation Strategies: Two-Part Model and PPML.

	(1)	(2)	(3)
Variable	Probit	Gamma-log GLM	PPML
TMI	2.011 *** (0.595)	0.602 (0.712)	1.835 ** (0.932)
TMI^2^	−0.415 *** (0.158)	−0.110 (0.161)	−0.408 * (0.243)
Control variables	Yes	Yes	Yes
Village FE	Yes	Yes	Yes
Log likelihood	−95.14	−686.51	−26564.60
Model fit statistic	Wald x^2^ = 65.28 ***	Deviance = 98.59	Wald x^2^ = 28.32 ***
Pseudo R^2^	0.262	-	0.400
N	186	103	196

Note: *** *p* < 0.01, ** *p* < 0.05, * *p* < 0.1. The Probit model reports coefficient estimates. The GLM and PPML models report coefficient estimates under a log-link specification. Heteroskedasticity-robust standard errors are reported in parentheses. The pseudo R^2^ reported for the Probit model is McFadden’s pseudo R^2^, while that reported for the PPML model is a McFadden-type pseudo R^2^ based on the pseudo-likelihood. Sample sizes vary across models. In the Probit model, some observations are dropped because of perfect prediction. The GLM uses only the subsample with positive food waste. The PPML model excludes observations corresponding to villages where all sampled households within the village had zero food waste.

## Appendix E

**Table A6:** Interaction Estimates for HPF and HDDS.

	(1)	(2)	(3)	(4)
Variable	HPF	AnyFW	HDDS	AnyFW
TMI	1.044 *** (0.285)		0.479 (0.684)	
TMI^2^	−0.240 *** (0.079)		−0.074 (0.175)	
cTMI×cHPF		0.128 *** (0.049)		
${cTMI}^{2}\times cHPF$		0.072 (0.056)		
cTMI×cHDDS				−0.012 (0.022)
${cTMI}^{2}\times cHDDS$				−0.011 (0.029)
cTMI		0.147 ** (0.074)		0.124 (0.079)
${cTMI}^{2}$		−0.154 *** (0.053)		−0.122 *** (0.043)
cHPF		−0.089 (0.061)		
cHDDS				0.065 (0.041)
Control variables	Yes	Yes	Yes	Yes
Village FE	Yes	Yes	Yes	Yes
Pseudo R^2^/R^2^	0.292	0.296	0.258	0.286
Overall significance statistic	F = 3.78 ***	Wald x^2^ = 62.16 ***	F = 2.78 ***	Wald x^2^ = 60.03 ***
Log likelihood	-	−90.80	-	−92.10
N	204	186	204	186

Note: *** *p* < 0.01, ** *p* < 0.05, * *p* < 0.1. The Logit model reports log-odds coefficients, and the OLS model reports linear regression coefficients. Heteroskedasticity-robust standard errors are reported in parentheses. The pseudo R^2^ reported for the Logit model is McFadden’s pseudo R^2^, while the OLS model reports the conventional R^2^ which represents the goodness of fit for ordinary least squares regression. The OLS model includes the full sample, whereas the Logit model excludes some observations due to perfect prediction.

## Appendix F

**Table A7:** Heterogeneity by Food Type.

	(1)	(2)	(3)	(4)
Variable	PFW	NPFW	AFW	VFW
TMI	1.242 * (0.690)	2.648 *** (0.710)	0.671 (0.607)	2.187 *** (0.774)
TMI^2^	−0.287 (0.179)	−0.469 ** (0.182)	−0.015 (0.158)	−0.438 ** (0.204)
Control variables	Yes	Yes	Yes	Yes
Village FE	Yes	Yes	Yes	Yes
Log likelihood	−247.06	−252.33	−129.06	−326.19
F-test	15.20 ***	14.95 ***	-	19.14 ***
Pseudo R^2^	0.199	0.119	0.147	0.131
Left-censored observations	132	137	175	106
N	204	204	204	204

Note: *** *p* < 0.01, ** *p* < 0.05, * *p* < 0.1. The Tobit estimates report average marginal estimates based on the expected observed values; heteroskedasticity-robust standard errors are reported in parentheses.
