# Supplementary material for: Transport Motorization and Household Food Waste in Rural China: Direct-Weighing Evidence of a Nonlinear Association
Source: Foods. 2026 Jul 25;15(15):2608. doi: 10.3390/foods15152608 (PMC13464130; doi:10.3390/foods15152608)
Supplement: Supplementary file 1 [file foods-15-02608-s001.zip › foods-4414147-supplementary.pdf]

Supplementary File S1

HOUSEHOLD FOOD CONSUMPTION AND NUTRIENT INTAKE QUESTIONNAIRE (Excerpt)

Translation note: This document is an author-prepared English translation. It was not a separate English-language form provided to participants. The items listed in this questionnaire are a subset of the full Shandong Province Rural Household Consumption and Nutrient Intake Special Survey Questionnaire, including only those that are analyzed in this paper.

Dear Sir/Madam,

This survey is part of a scientific research project on household food consumption and nutrient intake jointly conducted by the Institute of Geographic Sciences and Natural Resources Research, Chinese Academy of Sciences, and collaborating institutions. The survey is conducted anonymously, and the results will be used solely for academic research. We will adhere to the ethical standards of scientific research, and the information collected through the questionnaire will not be used for any other purpose. Please carefully read the questions in each section and tick the appropriate response or fill in the required information. Thank you sincerely for your cooperation.

1.How many usual residents currently live in your household? ____ persons

2.How many usual residents in your household are aged 60 years or above? ____ persons

3.How many usual residents in your household are under 18 years old? ____ persons

4.What is the approximate total capacity of the refrigerator currently used by your household? ____ liters

5.Does your household have any usual resident with diabetes?

A. Yes

B. No

6.What was the approximate gross income of your household in the past year?

A. Less than 10,000 yuan

B. 10,000-40,000 yuan

C. 40,000-80,000 yuan

D. 80,000-120,000 yuan

E. More than 120,000 yuan

7.What is the education level of the household head?

A. No schooling

B. Primary school

C. Junior middle school

D. Senior high school or technical secondary school

E. College degree or above

8.In the past three months, excluding the most recent three days before the survey, have you noticed or heard any information about food waste or food-waste reduction?

A. Never

B. Rarely

C. Occasionally

D. Often

E. Very often

9. Food purchases in the past week

Food Category	Purchase Frequency in the Past Week (A)	Purchase Location (B)	Distance (C)	Mode of Transport (D)
Vegetables				
Meat				
Rice				
Flour				
Corn flour				
cooked wheaten food				
Fruits				
drinks				

A: ① Twice or more per week ② Once per week ③ Once every half month ④ Once per month ⑤ Once per quarter ⑥ Once every six months ⑦ Once per year ⑧ Do not purchase ⑨ Once every two months

If option ① is selected, record the most recent purchase time, annual purchase frequency, and related information.

B: ① Convenience store ② Supermarket ③ Farmers' market ④ Specialized fruit or vegetable store ⑤ Grain and oil store ⑥ Mobile vendor ⑦ Online purchase ⑧ Self-owned or entrusted processing

C: ① Within 500 meters ② 500-3,000 meters ③ 3,001-5,000 meters ④ More than 5,000 meters

D: ① Walking ② Bicycle ③ Electric bicycle ④ Car ⑤ Public transport

Supplementary File S2

Dietary Consumption Record Form

Translation note: This document is an author-prepared English translation. It was not a separate English-language form provided to household survey volunteers.

1. Consumption Weighing Tracking Form

	flour and flour-based foods			rice			maize and cornmeal		
	Weight before cooking (g)	Weight of food discarded after meal (g)	Remarks	Weight before cooking (g)	Weight of food discarded after meal (g)	Remarks	Weight before cooking (g)	Weight of food discarded after meal (g)	Remarks
Day 1 Breakfast									
Day 1 Lunch									
Day 1 Dinner									
Day 2 Breakfast									
Day 2 Lunch									
Day 2 Dinner									
Day 3 Breakfast									

Day 3 Lunch									
Day 3 Dinner									

	beans and bean products			coarse grains			tubers		
	Weight before cooking (g)	Weight of food discarded after meal (g)	Remarks	Weight before cooking (g)	Weight of food discarded after meal (g)	Remarks	Weight before cooking (g)	Weight of food discarded after meal (g)	Remarks
Day 1 Breakfast									
Day 1 Lunch									
Day 1 Dinner									
Day 2 Breakfast									
Day 2 Lunch									
Day 2 Dinner									
Day 3 Breakfast									
Day 3 Lunch									
Day 3 Dinner									

	meat			eggs			milk		
	Weight before cooking (g)	Weight of food discarded after meal (g)	Remarks	Weight before cooking (g)	Weight of food discarded after meal (g)	Remarks	Weight before cooking (g)	Weight of food discarded after meal (g)	Remarks
Day 1 Breakfast									
Day 1 Lunch									
Day 1 Dinner									
Day 2 Breakfast									
Day 2 Lunch									
Day 2 Dinner									
Day 3 Breakfast									
Day 3 Lunch									
Day 3 Dinner									

	aquatic products			vegetables			fruits		
	Weight before cooking (g)	Weight of food discarded after meal (g)	Remarks	Weight before cooking (g)	Weight of food discarded after meal (g)	Remarks	Weight before cooking (g)	Weight of food discarded after meal (g)	Remarks

Day 1 Breakfast									
Day 1 Lunch									
Day 1 Dinner									
Day 2 Breakfast									
Day 2 Lunch									
Day 2 Dinner									
Day 3 Breakfast									
Day 3 Lunch									
Day 3 Dinner									

	snacks			drinks			edible oil		
	Weight before cooking (g)	Weight of food discarded after meal (g)	Remarks	Weight before cooking (g)	Weight of food discarded after meal (g)	Remarks	Weight before cooking (g)	Weight of food discarded after meal (g)	Remarks
Day 1 Breakfast									
Day 1 Lunch									
Day 1 Dinner									

Day 2 Breakfast									
Day 2 Lunch									
Day 2 Dinner									
Day 3 Breakfast									
Day 3 Lunch									
Day 3 Dinner									

	condiments		
	Weight before cooking (g)	Weight of food discarded after meal (g)	Remarks
Day 1 Breakfast			
Day 1 Lunch			
Day 1 Dinner			
Day 2 Breakfast			
Day 2 Lunch			
Day 2 Dinner			

Day 3 Breakfast			
Day 3 Lunch			
Day 3 Dinner			

2. Daily Separate Discard Recording Form

	flour and flour-based foods		rice		maize and cornmeal		beans and bean products		coarse grains		tubers	
	Weight (g)	Remarks	Weight (g)	Remarks	Weight (g)	Remarks	Weight (g)	Remarks	Weight (g)	Remarks	Weight (g)	Remarks
Day 1												
Day 2												
Day 3												

	meat		eggs		milk		aquatic products		vegetables		fruits	
	Weight (g)	Remarks	Weight (g)	Remarks	Weight (g)	Remarks	Weight (g)	Remarks	Weight (g)	Remarks	Weight (g)	Remarks
Day 1												
Day 2												

Day 3												

	snacks		drinks		edible oil		condiments	
	Weight (g)	Remarks	Weight (g)	Remarks	Weight (g)	Remarks	Weight (g)	Remarks
Day 1								
Day 2								
Day 3								

Supplementary File S3

FIELD CLASSIFICATION AND DIRECT-WEIGHING GUIDELINES

Source note: This author-prepared English summary is based on the survey instructions, enumerator-training content, and recording rules used in the 2017 fieldwork. It is supplied to document the field protocol and was not a separate English-language form provided to participating households.

S3.1 Operational decision rules

Decision point	Field rule applied
**Observation scope**	Record actual food disposal observed during the three-day household tracking window at the household consumption stage.
**Acquisition source**	Apply the same inclusion rule regardless of acquisition source. Food obtained through household production (including produce from home gardens and animal-source foods from household-raised poultry or livestock), gifts from others, household purchases, or other channels is included if discarded during the observation window.
**Source-specific identification**	The food-waste record did not retain the acquisition source of each discarded item as a separate field; therefore, food waste could not be disaggregated by acquisition source.
**Included material**	Count food that was originally edible, could reasonably have been consumed, and was discarded from household storage, immediately after breakfast, lunch, or dinner, or after being retained as leftovers but subsequently discarded without being consumed.
**Excluded material**	Exclude clearly inedible residues under the survey definition, including vegetable peels, soybean dregs, and bones. Food retained for later consumption is not counted unless it is subsequently discarded during the observation window.
**Waste stage**	Classify edible food discarded from household storage because it had expired or spoiled, together with meal leftovers retained after breakfast, lunch, or dinner but subsequently discarded without being consumed, as storage waste. Classify edible food discarded immediately after breakfast, lunch, or dinner as plate waste.
**Mixed leftovers**	Remove identifiable inedible parts before weighing. Separate edible components from inedible residues as far as possible, record the edible portion

Decision point	Field rule applied
	in the appropriate food category, and use the Remarks field where clarification is needed.
**Food category**	Use the 16-category classification framework: flour and flour-based foods; rice; maize and cornmeal; beans and bean products; coarse grains; tubers; meat; eggs; milk; aquatic products; vegetables; fruits; snacks; drinks; edible oil; and condiments.
**Recording frequency**	Record plate waste after breakfast, lunch, and dinner over two consecutive weekdays and one weekend day, yielding nine recorded meals per household. Record storage waste once per day.
**Weighing and units**	Use the uniform electronic scales supplied to field teams and record measured weight in grams. Convert cooked-food weights to raw-food-equivalent weights during data processing using the stated conversion procedures.
**Initial household stocks**	Include edible stored food when it is discarded during the observation window, even if acquired earlier. Do not interpret the measure as a full inventory cycle because stocks present at the start of the three days were not inventoried.
**Responsibility and quality control**	A trained enumerator—not the respondent household—makes the on-site classification. Enumerators received standardized instruction in classification, boundary judgement, weighing, and recording; the research team reviewed the completed waste records after fieldwork.

S3.2 Relationship between the two category systems

The eight-category transport module and the 16-category waste record are separate instruments. Supplementary File S2 contains the complete 16-category food-waste recording forms.The transport module collected category-specific purchasing frequency, location, distance, and transport-mode information. The transport-mode responses for the eight categories, weighted by the corresponding household consumption shares, were used to construct the TMI. The waste record used broader coverage to capture discarded edible food comprehensively. Accordingly, the presence of eight transport categories in Supplementary File S1 does not indicate that waste measurement was restricted to eight categories.

Transport-module categories: vegetables; meat; rice; flour; corn flour; cooked wheaten foods; fruits; and drinks.

Waste-record categories: flour and flour-based foods; rice; maize and cornmeal; beans and bean products; coarse grains; tubers; meat; eggs; milk; aquatic products; vegetables; fruits; snacks; drinks; edible oil; and condiments.
